# Effective and Green Removal of Trichloroacetic Acid from Disinfected Water

**DOI:** 10.3390/ma13040827

**Published:** 2020-02-12

**Authors:** Keren Trabelsi, Michael Meistelman, Rosaria Ciriminna, Yael Albo, Mario Pagliaro

**Affiliations:** 1 Department Chemical Engineering, The Center for Radical Reactions, Ariel University, Ariel 40700, Israel; trabelsi.keren@gmail.com (K.T.); michael.meistelman@gmail.com (M.M.); 2 Istituto per lo Studio dei Materiali Nanostrutturati, CNR, 90146 Palermo, Italy; rosaria.ciriminna@cnr.it

**Keywords:** halogenated by-products, drinking water, trichloroacetic acid, environmental catalysis

## Abstract

An innovative catalyst is reported for removing suspect carcinogen trichloroacetic acid (TCA) found in water after chlorination. SilverSil, a methyl-modified silica xerogel doped with Ag nanoparticles, shows remarkably high and stable activity as heterogeneous catalyst for the reductive dehalogenation of TCA with NaBH_4_ as reducing agent. Chloroacetic acid and acetic acid are the main products of the highly reproducible reductive dehalogenation. The low cost, high stability and ease of application of the SilverSil sol-gel catalyst to continuous processes open the route to the industrial uptake of SilverSil to free chlorinated waters from a probable human carcinogenic agent exerting significant genotoxic and cytotoxic effects.

## 1. Introduction

In 1974 Rook, a chemist at Rotterdam Waters, discovered that the reaction of natural organic matter in water with chlorine during water disinfection leads to the formation of haloforms as unwanted by-products [1]. Regardless of low concentration in water, where most disinfection by-products are poorly soluble, chlorination by-product compounds which also include haloacetic acids, haloacetonitriles and halocarbonyl compounds, are toxic and tend to accumulate in the food chain [2]. Hence, guideline values exist in most world’s countries, with adsorption on activated carbons being the main method used for the removal of volatile trihalomethanes [2]. For example, the 0.1 mg/L guideline value for trichloroacetic acid (Cl_3_CCOOH) in drinking-water was first suggested by the World Health Organization (WHO) in 1993 as “provisional value” due to “the limitations of the available toxicological database” [3].

Trichloroacetic acid (TCA) is a suspect human carcinogen exerting significant genotoxic and cytotoxic effects, which accumulates in the body [4]. Analysis of TCA in urine samples from a reference population of 402 adults in the United States of America in 2003 showed presence of the acid in 76% of the samples analysed, with a 90th percentile concentration of 23 μg/L [5]. Almost all (81%) residents in urban areas, and a vast majority (62%) of residents in rural areas had urinary TCA, though with significant higher concentrations for residents in urban areas.

The high toxicity and persistency of TCA have prompted extensive research concerning its degradation to environmentally safe products. Different technologies have been developed including biodegradation [6], electrochemical methods [7,8,9,10], radiation induced methods [11,12], photochemical processes [13], photosonochemical degradation [14] and chemical methods based on advanced reduction process based on carboxyl anion radical [15], as well as the removal of TCA (and other disinfection by-products) by granular and biological activated carbons [16,17]. 

However, despite the different suggested technologies, no water treatment method for TCA has been recommended by a regulatory body. According to the *World Health Organization Guidelines for Drinking-Water Quality* published in 2004 “concentrations may be reduced by installing or optimizing coagulation to remove precursors and/or by controlling the pH during chlorination” [3].

**Scheme 1:**
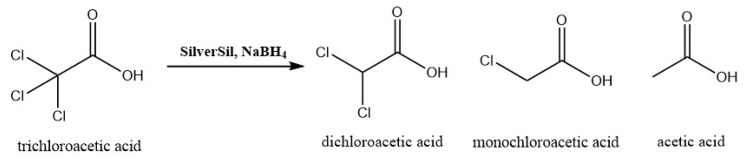
Reductive dehalogenation of trichloroacetic acid (TCA) with sodium borohydride mediated by SilverSil.

We now report that SilverSil, a methyl-modified silica xerogel doped with Ag nanoparticles of exceptionally high antibacterial activity [18], also shows remarkably high and stable activity as heterogeneous catalyst for the reductive dehalogenation of TCA dissolved in water in high concentration (229 µg/L) using green and safe NaBH_4_ as reducing agent (Scheme 1). 

## 2. Materials and Methods

### 2.1. Materials 

Trichloroacetic acid was obtained from Alfa Aesar. Tetraethyl orthosilicate (TEOS), methyltriethoxysilane (MTEOS) and silver nitrate were purchased from Aldrich. NaBH_4_ was bought from Strem Chemicals. All aqueous solutions were prepared from deionized water purified by a Millipore Milli-Q setup (Darmstadt, Germany) with a final resistivity of >10 MΩ/cm.

### 2.2. Syntheses and Characterization

The preparation of SilverSil organically modified silicas (ORMOSILs) by sol-gel hydrolytic co-polycondensation of TEOS and MTEOS in the presence of AgNO_3_ has been described elsewhere [18]. The composition of the xerogels used throughout this study is summarized in Table 1.

In a typical preparation of the 70:30 SilverSil (70 mol percent TEOS and 30 mol percent MTEOS), 6.0 mL of aqueous HNO_3_ solution (0.2 M) was added dropwise to a solution consisting of TEOS (8.8 mL), MTEOS (3.4 mL) and 13.3 mL of ethanol. After stirring for 10 min, 125 µL of (3-aminopropyl)triethoxysilane (APS) were added to the mixture. Hence, an aliquot (5.0 mL) of a 6.36 mM aqueous solution of AgNO_3_ was added dropwise, after which water (5.0 mL) and aqueous ammonia (0.01 M, 5.0 mL) were added to promote the hydrolytic polycondensation. A wet gel was quickly obtained which was dried at room temperature for approximately two weeks until a constant weight of the dry matrix was obtained. The xerogel was crushed into a powder with a pestle, after which the powder was treated with aqueous NaBH_4_ (0.03 M, 100.0 mL) to promote reduction of the entrapped Ag^+^ ions. At this stage the xerogel changed colour from white to light yellow characteristic to silver nanoparticles (NPs). The material obtained was air-dried again. The yellow xerogel was crushed in a mortar and the powder used as such in subsequent catalytic dehalogenation reactions of TCA.

The textural properties of the SilverSil ORMOSILs were determined from N_2_ adsorption–desorption isotherms measured at liquid nitrogen temperature using a NOVA 3200E Quantachrome analyzer. The specific surface area was calculated from the linear section of the Brunauer–Emmett–Teller (BET) plot. The UV-vis spectra were measured using a Varian Cary UV Bio 50 spectrophotometer.

### 2.3. Catalytic Tests

In a typical catalytic run, 2.8 mL of 0.010 M TCA was added to the suspension of 0.50 g of the SilverSil taken in 14.4 mL of deionised water in a 50 mL beaker kept at room temperature. The mixture was then stirred for a few minutes. A 2.8 mL aliquot of 0.060 M of aqueous NaBH_4_ was thus added, and the resulting solution further stirred for 2 h. After completion of the reaction the catalyst was recovered by filtration and the filtrate analysed by HPLC. The catalyst was washed extensively with water, dried in air at room temperature and reused as such in subsequent reaction runs under the same conditions. Each result reported is the average of at least three independent experiments. The error limit for the analytical results is ±5%.

Under said reaction conditions, no dehalogenation was detected without added catalyst (blank test). In addition, in the absence of NaBH_4_ but in the presence of a catalytic amount of SilverSil no reduction was observed (blank test). 

The dehalogenation of TCA was monitored by HPLC using a Dionex Ultimate 3000 chromatograph equipped with VWD (variable wavelength detector) flow cell in line with ISQ quadrupole mass spectrometer (MS, Thermo Fisher Scientific, Austin, TX, USA) The Ultimate 3000 chromatograph was equipped with Diode Array Detector by Thermo (λ = 210 nm) and a 4.6 mm × 150 mm HPLC column comprised of 5 µm organosilica microparticles (Agilent, Eclipse XDB-C18). The eluent, an aqueous H_2_O:CH_3_CN = 90:10 mixture kept at pH 2.0 due to the presence of 0.1 wt% H_3_PO_4_, was flowed at 1.0 mL/min rate.

## 3. Results and Discussion

### 3.1. Catalyst Characterization

**Figure 1:**
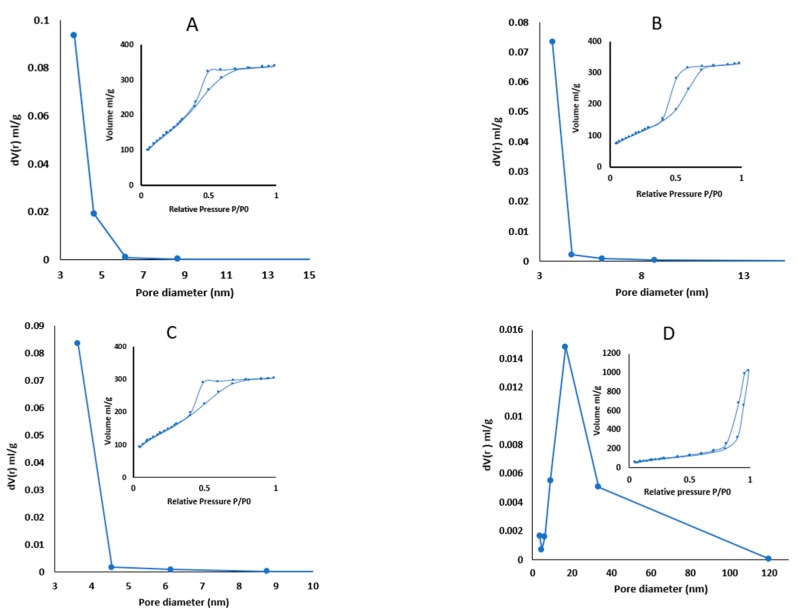
Pore size distribution of different SilverSil xerogels: (**A**) (90:10) (inset Brunauer–Emmett–Teller (BET) isotherm of the same matrix), (**B**) (70:30), (**C**) (50:50) and (**D**) (30:70).

The N_2_ adsorption–desorption isotherms (Figure 1) are type IV curves characteristic of glassy mesoporous materials with uniform pores.

In general, the SilverSil xerogels have narrow Barrett–Joyner–Halenda (BJH) pore size distribution, with average pore diameters nearly 4.0 nm for all compositions, except for the 30:70 SilverSil xerogel having an average pore diameter of 20 nm. The Brunauer–Emmett–Teller (BET) surface area and pore volume of the SilverSil matrices are summarized in Table 1.

### 3.2. Catalytic Activity Towards Dehalogenation of TCA 

**Figure 2:**
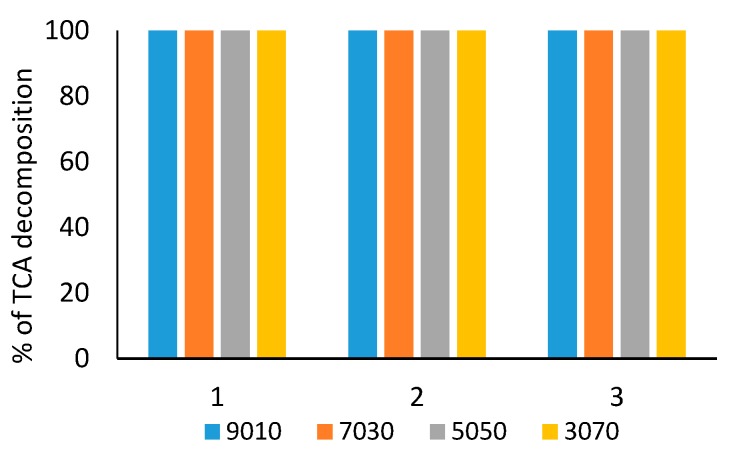
Decomposition of TCA after 2 h reductive dehalogenation over different SilverSil xerogels with NaBH_4_ as reducing agent in 6:1 molar ratio, [TCA] = 1.4 × 10^−3^ M, [BH_4_^−^] = 8.4 × 10^−3^ M.

Figure 2 shows that TCA is entirely decomposed over all the tested SilverSil xerogels, going from relatively hydrophilic SilverSil 90:10, through hydrophobic SilverSil 30:70. The same plot shows that all catalysts, tested in 3 consecutive reaction runs, exhibit excellent catalytic stability being fully reusable with no loss of activity.

**Figure 3:**
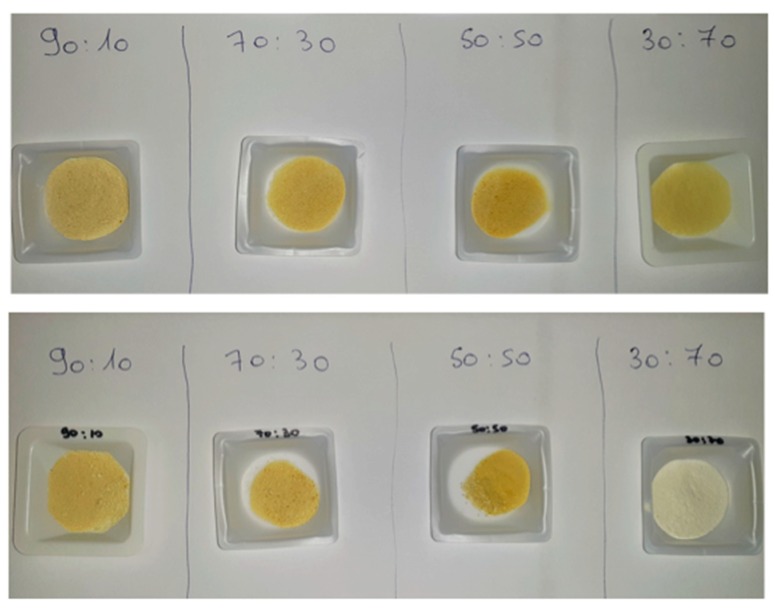
Visual appearance of different SilverSil xerogels prior (**top**) and after TCA dehalogenation reaction with NaBH_4_ as reducing agent in 6:1 molar ratio (**bottom**).

The visual appearance of the SilverSil catalysts prior and after 3 consecutive reactions runs is shown in Figure 3.

Catalyst decolouration observed only for the 30:70 SilverSil xerogel of larger porosity points to likely surface modification of the nanoparticle upon the interaction with the reactants in solution (BH_4_^-^ and TCA) within the much larger porosity of the latter xerogel. 

**Figure 4:**
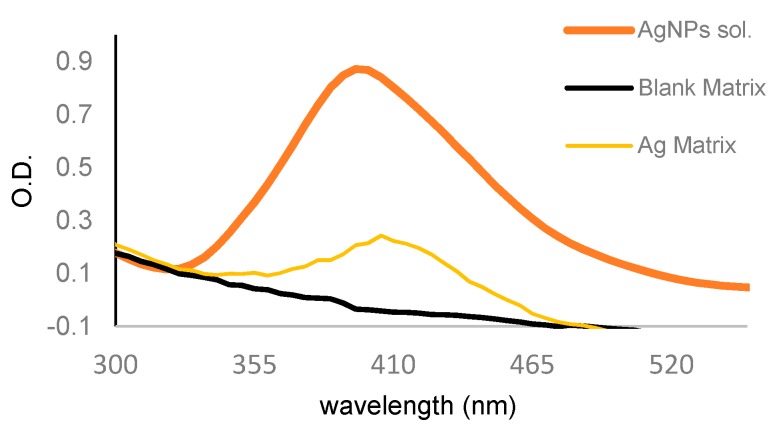
UV-vis absorption spectrum for the 30:70 SilverSil xerogel, blank matrix and silver nanoparticles.

We remind that the yellow colour of ORMOSIL glasses functionalized with silver (or gold) nanoparticles is due to the surface plasmon resonance (SPR) of the entrapped metal nanoparticles [19]. Figure 4 displays the UV-vis spectra for Ag NPs suspension (about 20 nm in size), for 30:70 SilverSil, and for the blank matrix.

The reduced intensity in the light absorption in the 30:70 xerogel after 3 reaction runs may be due to formation of surface-modified Ag^0^ nanoparticles, due to the action of both BH_4_^-^ and TCA hydrophobic moieties adsorbed and concentrated at the surface of the sol-gel cages entrapping the silver NPs. ORMOSILs indeed are well known to act as chemical sponges, adsorbing and concentrating reactants are their inner surface [20].

**Figure 5:**
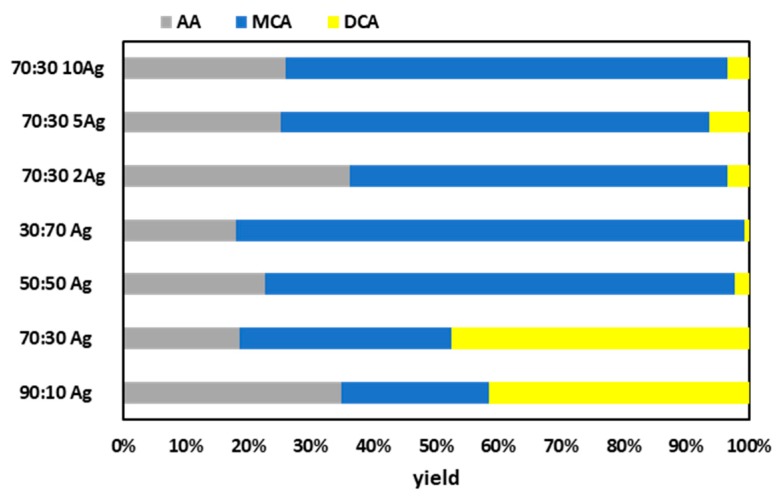
Product distribution after 2 h reaction in TCA reductive dehalogenation of different SilverSil xerogels, and over the same 70:30 xerogel in increasing amounts, with NaBH_4_ as reducing agent in 6:1 molar ratio, [TCA] = 1.4 × 10^−3^ M, [BH_4_^-^] = 8.4 × 10^−3^ M.

Figure 5 displays the large effect of the ORMOSIL composition on the product distribution. For all SilverSil compositions the TCA decomposition is complete. The more hydrophilic SilverSil 90:10 and 70:30 xerogels showed the highest amount of dichloroacetic acid (DCA) and acetic acid (AA) production. The lowest DCA formation is obtained at the surface of the more hydrophobic (50% and 70% methyl-modified) silica glasses.

These results show once again evidence of the key role played by the ORMOSIL surface hydrophilic-lipophilic balance (HLB) on the catalytic activity of the functionalized ORMOSIL [21]. The 70% methyl-modified xerogel 30:70 SilverSil is able to completely degrade TCA almost entirely to monochloroacetic acid (MCA, often called chloroacetic acid) and to fully de-halogenated product, acetic acid. Unlike DCA and TCA, monochloroacetic acid is not carcinogenic and does not show mutagenic action [22]. In 2004, the WHO recommended a guideline value for MCA of 20 µg/L in drinking-water, based on an allocation of 20% of the tolerable daily intake assuming a 60-kg adult ingesting 2 litres of drinking water per day [23].

**Figure 6:**
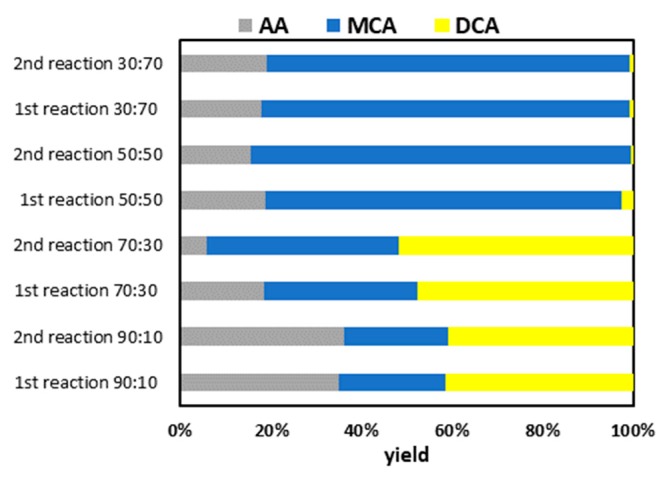
Product distribution after 2 h reaction in TCA reductive dehalogenation of different SilverSil xerogels with NaBH_4_ as reducing agent in 6:1 molar ratio in two reaction runs, [TCA] = 1.4 × 10^−3^ M, [BH_4_^−^] = 8.4 × 10^−3^ M.

Remarkably, the high reproducibility of the TCA catalytic reduction over the SilverSil xerogels is demonstrated by the almost unvaried product distribution obtained in two catalytic runs carried out in batch (Figure 6). 

**Figure 7:**
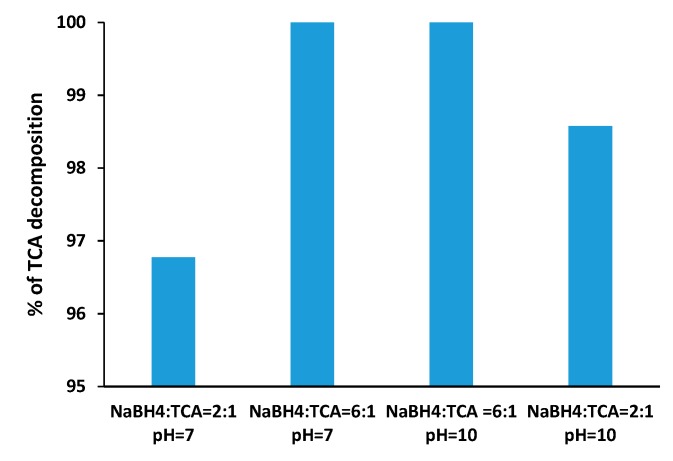
Decomposition of TCA after 2 h reductive dehalogenation over 70:30 SilverSil xerogel in TCA reductive dehalogenation at different pH and with NaBH_4_ as reducing agent in 2:1 and 6:1 molar ratio, [TCA] = 1.4 × 10^−3^ M.

Finally, Figure 7 presents the effect of TCA:NaBH_4_ molar ratio and the pH on the TCA decomposition yields. As expected, higher levels of TCA reduction were obtained for higher NaBH_4_ concentrations, with complete TCA decomposition taking place also at pH 7 provided that borohydride is used in 6:1 molar ratio with respect to the substrate.

## 4. Conclusions

In conclusion, we have discovered that 30:70 SilverSil, a 70% methyl-modified silica xerogel doped with Ag nanoparticles, shows remarkably high and stable activity as heterogeneous catalyst for the reductive dehalogenation of trichloroacetic acid (a suspect human carcinogen) with NaBH_4_ as reducing agent. Non carcinogenic and non mutagenic chloroacetic acid is, along with non toxic acetic acid, the main product of the reductive dehalogenation. The excess borohydride is rapidly decomposed to boric acid by the powerful catalytic activity of the Ag nanoparticles. Found in virtually all vascular plants (where boron exists in the equilibrium between boric acid [B(OH)_3_] and borate anion [B(OH)_4_^−^), boric acid (B(OH_3_) byproduct of the water treatment proposed in the aforementioned processes is a weak Lewis acid (pK*_a_* = 9.25) mostly undissociated at neutral pH [23]. The compound naturally occurs in several fruit-derived beverages including wine and cider.

Following the discovery of ORMOSIL-entrapped Au nanoparticles in the reductive dehalogenation of mono- and tri-bromo acetic acids with sodium borohydride [24], the high activity of sol-gel entrapped silver nanoparticles in the reductive dehalogenation of toxic bromoacetic acid (the most toxic of the regulated trihalomethanes and haloacetic acids), formed during water chlorination was reported in 2017 [25]. This means, in principle, that by using a single cartridge filled with 70% methyl-modified mesoporous SilverSil xerogel it will be possible to remove from disinfected waters both brominated chlorination by-products and trichloroacetic acid. 

Future work aimed at evaluating the degradation of more highly brominated or Br/Cl acetic acids will be reported soon. In conclusion, the low cost, high stability and ease of application of the SilverSil sol-gel catalyst to continuous processes open the route to the industrial uptake of SilverSil to remove a suspected human carcinogenic agent exerting significant genotoxic and cytotoxic effects from chlorinated waters. ORMOSILs functionalized with different dopant species exhibit uniquely high mechanical, thermal and chemical stability which, along with their high functional activity and lack of toxicity, originates manifold applications [20,26]. Hydrothermally stable ORMOSIL-based membranes highly resistant to NaClO in solution, for instance, are already commercialized for enhanced, low energy separation processes [27]. Further research will address the development of one such column reactor functionalized with an optimal SilverSil coating for the effective removal of haloacetic acids from chlorinated water at real plants.

## Figures and Tables

**Table 1:** Composition of the different SilverSil materials.

SilverSil	TEOS (mol%)	MTEOS (mol%)	Ag(0) Load (mmol Ag)	Specific Surface Area (m^2^/g)	Specific Pore Volume (cm^3^/g)
90:10 Ag	90	10	0.0325	394.036	0.9811
70:30 Ag	70	30	0.0325	582.475	0.9918
50:50 Ag	50	50	0.0325	506.046	0.9907
30:70 Ag	30	70	0.0325	308.583	1.581
70:30 5Ag	70	30	0.1625	-	-
70:30 10Ag	70	30	0.325	-	-
